# With a grain of salt: Sodium elevation and metabolic remodelling in heart failure

**DOI:** 10.1016/j.yjmcc.2021.08.003

**Published:** 2021-08-08

**Authors:** Dunja Aksentijević, Michael J. Shattock

**Affiliations:** a William Harvey Research Institute, Centre for Biochemical Pharmacology, Barts and the London School of Medicine and Dentistry, Queen Mary University of London, Charterhouse Square, London, UK; b School of Cardiovascular Medicine and Sciences, British Heart Foundation Centre of Research Excellence, King’s College London, The Rayne Institute, St Thomas’ Hospital, London, UK

**Keywords:** Sodium, Cardiac metabolism, Heart failure, Sodium pump, Energetics

## Abstract

Elevated intracellular Na (Na_i_) and metabolic impairment are interrelated pathophysiological features of the failing heart (HF). There have been a number of studies showing that myocardial sodium elevation subtly affects mitochondrial function. During contraction, mitochondrial calcium (Ca_mito_) stimulates a variety of TCA cycle enzymes, thereby providing reducing equivalents to maintain ATP supply. Na_i_ elevation has been shown to impact Ca_mito_; however, whether metabolic remodelling in HF is caused by increased Na_i_ has only been recently demonstrated. This novel insight may help to elucidate the contribution of metabolic remodelling in the pathophysiology of HF, the lack of efficacy of current HF therapies and a rationale for the development of future metabolism-targeting treatments. Here we review the relationship between Na pump inhibition, elevated Na_i_, and altered metabolic profile in the context of HF and their link to metabolic (in)flexibility and mitochondrial reprogramming.

## 1 Introduction

Heart failure (HF) imposes an enormous worldwide medical and economic burden. With few effective treatments available, heart failure (HF) affects over 64 million people worldwide carrying the annual death toll of 17.5 million lives [1]. Despite the advancements in diagnostic tools and therapies, with an ever-ageing population it’s prevalence is on continuous rise. The current COVID-19 pandemic has added to this chronic disease burden as the patients with underlying cardiovascular disease face significantly poorer prognosis [2–6]. Thus, there is a clear and rapidly increasing requirement for improved understanding of fundamental cellular mechanisms in HF which can, in turn, help the development of improved treatments and innovative diagnostic techniques.

Numerous molecular mechanisms have been proposed that could contribute to the development of HF and these include an energy deficit following metabolic reprogramming. In a series of precisely regulated enzymatic reactions, heart muscle highly efficiently converts chemical into mechanical energy [7]. This fact is easily obscured by the complexities of myocardial anatomy, haemodynamics and coronary flow. Despite myocardial metabolism and function being inseparably linked, substrate metabolism as a paradigm for the development of novel HF therapies has been mostly overlooked [8]. In addition to the changes in cardiac metabolism, alterations in excitation–contraction (*E*-C) coupling including Na_i_ elevation are characteristic features of pathological cardiac remodelling and underpin contractile dysfunction in HF.

## 2 Intracellular Na regulation

In the healthy mammalian heart, cytoplasmic Na, Ca, and H concentrations are lower than their electrochemical equilibrium values. In the myocardium of most large animals the intracellular Na concentration is typically around 8-10 mM [9–11] (Table 1). In the murine heart (rats and mice) Na_i_ is reported to be significantly higher at 10-20 mM (reviewed in [9]) (Table 1). Due to differences in experimental methodologies, absolute values of measured Na_i_ may vary (Table 1). This elevated intracellular Na is associated with a range of other physiological adaptations including a higher heart rate, a shorter action potential and EC-coupling that is less dependent on transarcolemmal Ca flux and more dependent on intracellular Ca cycling [17]. In murine species, these adaptations appear to contribute to a relatively flat force-frequency relationship and the phenomenon of post-rest potentiation [12].

**Fig. 1:**
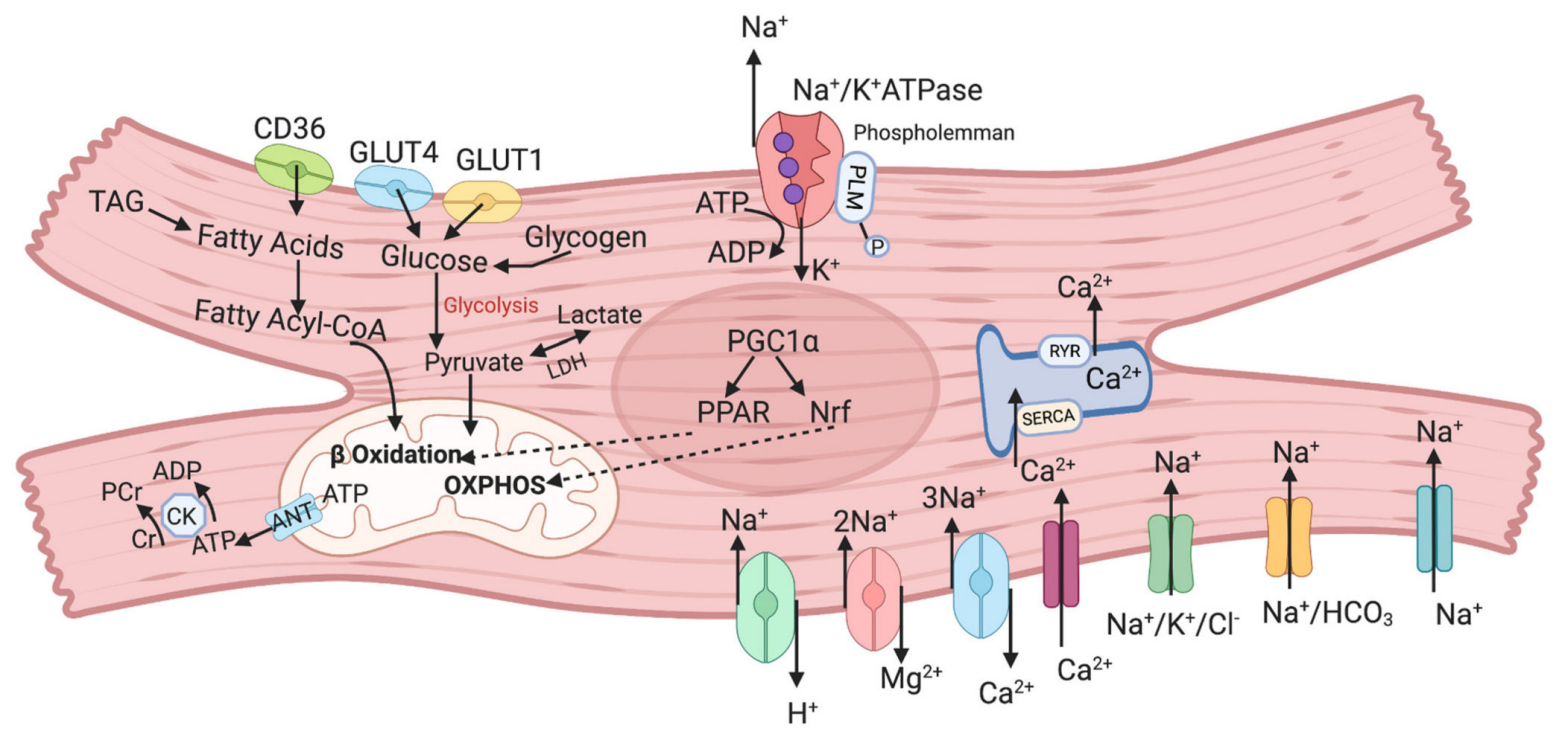
Myocardial ATP synthesis and Na_i_ influx/efflux pathways GLUT-glucose transporter (GLUT1 and GLUT4), CD36-fatty acid transporter, TAG-triacylglycerol, LDH-lactate dehydrogenase, CK-creatine kinase, PCr-phosphocreatine, Cr-creatine, ANT-adenine nucleotide translocase, RYR-ryanodine receptors, SERCA-Ca-ATPase in sarcoendoplasmic reticulum.

The low intracellular Na is maintained by the activity of the Na/K ATPase or Na/K pump (NKA). The energy invested by the cell in driving the Na/K ATPase, and maintaining this high transmembrane electrochemical gradient, creates a ‘battery’ that the cell then exploits to drive ions, substrates and amino acids into (symports or co-transporters) or out of (antiports or exchangers) the cell (Fig. 1). Each of these transmembrane transporters will tend to dissipate the Na gradient. As the Na/K ATPase is the only quantitatively significant efflux pathway, its activity, and capacity, needs to exceed the sum total of all these influx pathways combined (Fig. 1) [13].

The principal regulator of the activity of Na/K ATPase (NKA) is phospholemman (PLM) and plays a crucial role in regulation of cardiomyocyte contractility. NKA is a transmembrane protein consisting of *α* and *β* subunits found in the peripheral sarcolemma as well as T-tubules. α subunit is a highly conserved 110 kDa non-glycosylated protein. On the inner membrane it contains the ATPase domain and on the outer membrane ion-binding and ouabain-binding domains. β subunit is a ~55 kDa glycoprotein [14]. The Michaelis-Menten constant (K_m_) for ATP for the catalytic site is disputed, but is estimated to be *<<*1 mM [14]. In addition to ATP catalytic site, NKA also possess a low-affinity allosteric ATP-binding site. Both subunits comprise a family of isoforms whose expression varies during different stages of development, between atria and ventricles as well as in pathophysiological states including HF [14,15].

**Fig. 2:**
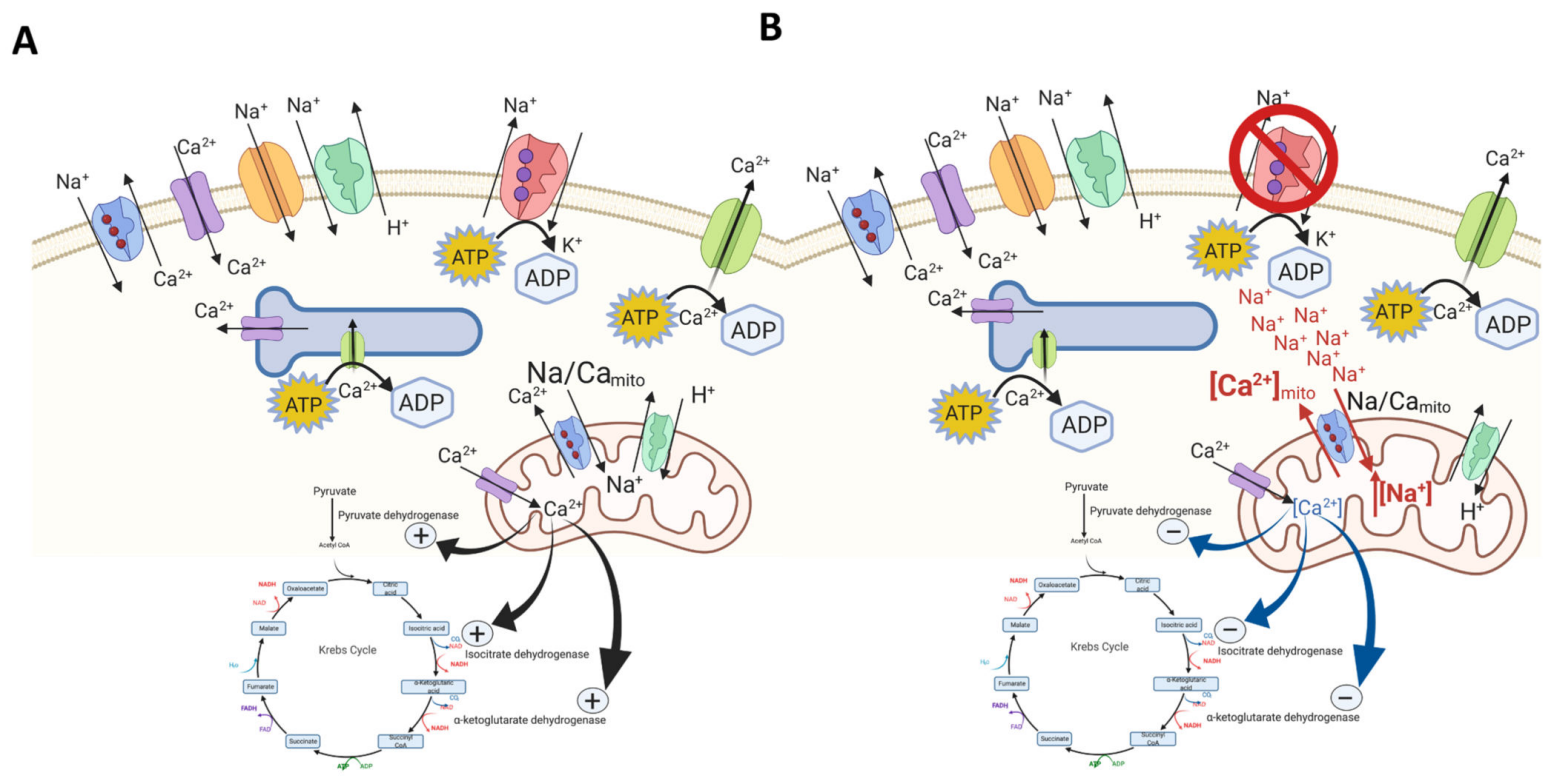
A) Mitochondrial Na/Ca transport and its relationship with mitochondrial ATP production B) Impact of Na_i_ elevation on mitochondrial metabolism via mitochondrial Na/Ca exchange.

NKA activity is strongly influenced by intracellular Na as the *K*_m_ for activation typically sits close to resting Na_i_. The *K*_m_ for NKA (and hence the prevailing Na_i_) is itself dynamically regulated by its accessory protein phospholemman (PLM) (FXYD1) which exerts a tonic inhibition on NKA [16]. This inhibition is relieved, and the *K*_m_ for Na reduced, by the phosphorylation of the cytoplasmic tail of PLM - principally by Protein Kinases A and C (Fig. 1) [17]. PLM phosphorylation is necessary for the active control of Na_i_ during sudden changes in heart rate or during disease and plays a vital role in Na regulation during ‘flight or fight’ and adrenergic simulation [18]. During the normal cardiac contractile cycle, NKA hydrolyses ATP to power the transport of Na and K across cell membranes. The stoichiometry of 3 Na extrusion in exchange for 2K import accompanied by the hydrolysis of 1 ATP, is strictly maintained. Furthermore, due to the sarcolemmal localization as well as high ATP demand required for its activity, NKA has been associated with preferential use of glycolytically-derived ATP [19–21]. Previous studies have suggested that cytosolic Na can also profoundly influence the feed-forward coupling between mechanical contraction and mitochondrial Ca-dependent ATP production (Fig. 2) [12,22]. Nonetheless, until recently the extent to which the mismatch in myocardial ATP supply–demand could arise as the consequence of elevated Na_i_ remained unclear.

## 3 Energy metabolism in the healthy heart

The heart has an enormous energy demand—it burns through 6 kg of ATP daily, consuming 2% of its total energy reserves per beat and turning over its total ATP pool in <1 min [23–26]. Despite this continuous dependence on ATP, its capacity to store ATP is miniscule: a 300 g human heart stores 30 mg ATP compared with the ATP utilization demand of 30 mg/s to sustain baseline cardiac function [27]. Therefore, it is predominantly reliant on aerobic metabolism for a continuous supply of ATP to fuel its mechanical work (Fig. 1). Mitochondria which occupy third of the cardiomyocyte volume account for more than 90% of generated ATP [28]. The large myocardial ATP demand is mainly related to EC coupling energy-dependant processes. Approximately 70–75% of total ATP turnover is used for force generation powering cardiac output, and the residual 25-30% used for the maintenance of basal metabolism [29,30]. In terms of force generation and ion regulation in the beating heart, it is estimated that the actomyosin ATPase accounts for 57%, SERCA (sarcoendoplasmic reticulum Ca ATPase) 11% and NKA for 7% of total ATP expenditure [29,31].Glucose, lactate, free fatty acids (FFA), ketone bodies and, under rare circumstances, amino acids, compete as catabolic substrates in order to meet constantly varying myocardial ATP demand [32]. This renders the healthy adult heart a “metabolic omnivore” and enables high degree of fuel flexibility [32]. Metabolic substrate selection is dynamic and in addition to changes in myocardial workload it is driven by O_2_ concentration and substrate availability [7,9].

The adult heart in vivo converts chemical energy predominantly stored in FFAs (60–90%) and pyruvate (10–40% derived from carbohydrates glucose and lactate) into contractile work [28]. However, the fate of metabolic substrates contributing to myocardial ATP provision is ultimately governed by multiple physiological factors: ATP demand, O_2_ supply/availability, availability and the type of carbon substrate heart is exposed to, hormonal influences, transcriptional, translational, and posttranslational control of the various components of metabolic pathways [33]. Combination of these factors ensures careful matching of myocardial ATP supply and demand beat-to-beat.

In order to avoid ATP waste and overall energetic inefficiency, the balance between carbohydrate and FFA utilization is carefully regulated by the Randle cycle (glucose-fatty acid cycle) [34]. Despite the complexity of converging metabolic pathways, myocardial ATP generation can be broken down into four principal stages. Metabolic substrate delivery (Stage 1), substrate selection, uptake and oxidation (Stage 2) to generate acetyl-CoA for TCA cycle entry (Stage 3). The 4th stage is by far the most important mechanism for aerobic ATP biosynthesis. It consists of two coupled processes: electron transport and oxidative phosphorylation (OXPHOS). Under aerobic conditions, mitochondrial OXPHOS accounts for ~90% ATP synthesis whilst the O_2_ supply-independent substrate level phosphorylation accounts for the residual ~10% [33]. In addition to O_2_ availability, mitochondrial OXPHOS is contingent on the availability of reducing equivalents (H^+^ and electrons) which are transferred from various energy-providing substrates to the mitochondria by the reduced forms of nicotinamide adenine dinucleotide (NAD—H^+^) and flavin adenine dinucleotide (FADH-H^+^), generated by dehydrogenase reactions that occur in the stepwise degradation of energy-providing substrates. Kreb’s cycle (TCA cycle) plays the role of the central metabolic hub and reducing equivalent provider by converging multiple metabolic pathways as the acetyl Co-A entering the Krebs cycle originates from the plethora of metabolic substrates (FFA, carbohydrates, amino acids and ketone bodies). Electron transport involves oxidation of NADH and FADH_2_ accompanied by the transport of electrons through a chain of oxidation/reduction reaction involving cytochromes (ETC) until donated to O_2_. The transport of electron drives H pumps in complexes I, III and IV. H are extruded from the mitochondrial matrix leading to matrix side of the membrane becoming negatively charged. This difference in electrochemical potential provides the energy for ATP synthesis when H return to the matrix through the F_0_ proton channel, thereby driving F1 ATP synthase (complex V) (Fig. 1). Upon synthesis, adenine nucleotide translocase (ANT) mediates ADP-ATP exchange across the inner mitochondrial membrane. This process initiates further cytosolic propagation of ATP/ADP disequilibria mediated by enzymes creatine kinase and adenylate kinase. Phosphotransfer therefore ensures ATP delivery from mitochondrial sites of synthesis to cytosolic ATP sinks. (Fig. 1) [35].

## 4 Na pump and regulation of Na_i_ in heart failure

Many studies of the failing myocardium report significantly elevated intracellular Na concentrations (reviewed in [9]) and, in part, this may be mediated by changes in NKA expression, activity and PLM phosphorylation [36]. The pathological consequences of elevation of intracellular Na include cellular Ca overload, a negative force–frequency relationship, impaired relaxation and arrhythmias [37]. While an increase in Na influx may be an important component of elevated Na_i_ [11], each individual influx pathway (such as Na/H exchanger, or slowly inactivating Na channel current) is quantitatively small in comparison to the NKA capacity. Hence, the reduction of NKA pump function, and/or expression, and PLM dephosphorylation, may be quantitatively more significant [16,18,36–38].

Originally identified in the early 20th century, myocardial energy starvation hypothesis has been widely accepted paradigm in HF. It postulates that chronic metabolic perturbations precede, initiate and maintain contractile dysfunction in HF [26]. Advances in technologies including use of nuclear magnetic resonance spectroscopy (NMRs) for pioneering human cardiac studies have improved mechanistic insights into “engine out of fuel” hallmark of HF and helped to classify alterations causing energetic deficit into those related to key steps of cardiac metabolism: substrate utilization, intermediary metabolism and energy reserve [39]. Decades of cardiac metabolism research have further developed and refined our understanding of the detrimental metabolic remodelling that characterizes HF. This helped to formulate our contemporary definition of metabolic perturbation in HF as a sum of chronic metabolic inefficiencies and lack of metabolic flexibility including alterations of intermediate substrate metabolism and oxidative stress, rather than an ATP deficit per se (reviewed in [40]).

Upon acute stress, in order to meet its ATP demand, heart readily shifts its “glucose-fatty acid cycle” to dominant carbohydrate catabolism [41]. When the heart is subject to persistent stress, such as chronic haemodynamic overload, it also reactivates foetal gene expression programme [42]. Thus, the switch from adult to foetal metabolic phenotype leads to extensive metabolic remodelling [32,43,44]. Even if initially adaptive, this switch ultimately leads to a loss of insulin sensitivity and consequent loss of metabolic flexibility [45]. The onset of the substrate switch as well as the stage at which it could be therapeutically targeted is subject to debate. Some studies suggest that cardiac energetics is only impaired during advanced stages of HF (ATP <30–40%) with ATP levels maintained during the initial stages of metabolic remodelling [46–50]. Nevertheless, series of seminal in vivo ^31^P NMRs studies [26,51–54] have helped to identify the reduction of the myocardial energy reserve defined as phosphocreatine-to-ATP ratio (PCr/ATP of <1.6) led to 44% increase in death from cardiovascular causes vs 5% of DCM patients with a PCr/ATP of >1.6. Collectively, these pioneering ^31^P NMRS studies helped to establish cardiac energetics as a powerful and reliable prognostic indicator in HF [55]. Furthermore, there have also been many preclinical and clinical studies describing the role of mitochondrial dysfunction: mitochondrial misalignment, reduced density, aggregation, disorganized cristae and membrane disruption in hypertrophy and HF [56–58]. Dysfunctional mitochondrial ETC in relation to ATP synthesis was shown to intensify ROS damage of proteins, lipids and DNA leading to cardiomyocyte loss [57].

The relationship between NKA function and metabolism is bidirectional. That is, as a significant consumer of ATP and the main determinant of Na_i_, NKA both responds to, and influences, metabolism. As an energy-dependent pump it is reasonable to hypothesise that declining ATP concentrations in the failing or ischaemic heart might compromise ion transport. In ischaemia, both PCr and ATP fall precipitously and, in the failing heart, the substrate switch from fatty acids to glucose leads to a decline in cytosolic PCr/ATP reserve limiting the ATP supply. However, even during severe metabolic stress, Na_i_ rises at a time when the total ATP concentration greatly exceeds the *K*_m_ for the pump (~0.1–0.8 mmol/l) and the Δ*G* of ATP exceeds that required for NKA activity (~44 kJ/mol) [59]. In hypoxia and ischaemia, the accumulation of cytosolic inhibitors of NKA activity, and redox changes may inhibit ion transport long before ATP supply becomes limiting [59]. There is, therefore, little evidence to suggest that a failure of metabolism and energy supply limits NKA activity. However, there is accumulating evidence for the contrary; that is, NKA inhibition and the associated increase in Na_i_ can directly influence mitochondrial metabolism [43,60]. In addition, both NKA and SERCA have been shown to preferentially depend on glycolytic metabolism [61] and, in the failing heart, the switch to a glycolytic phenotype could reflect changes in their activity an attempt to maintain ion homeostasis [19,62,63].

## 5 Metabolic remodelling and myocardial Na_i_ elevation: a causality dilemma

Despite the extensive evidence for the concomitance of remodelled metabolism and elevated Na_i_ in HF, studies investigating their interaction are scarce and mostly limited to isolated organelles. Increase in cytosolic Na (12.5 mM to ≥25 mM) was shown to reduce state 3 respiration in isolated rat mitochondria potentially impacting ATP synthesis [64]. Other isolated mitochondria studies have shown that extra-mitochondrial Na addition (1–10 mM) led to a dose-dependent decrease in OXPHOS which was reversed by addition of Ca or by diltiazem inhibition of Na/Ca_mito_ (NCLX) exchanger [65].

^31^P-NMR spectroscopy assessment of superfused mitochondria embedded in agarose beads showed that 3–30 mM increase in Na significantly reduced ATP synthesis, particularly in type 2 diabetic mitochondria [66]. Collectively, these studies suggested that supra-physiological Na elevation leads to abnormalities in OXPHOS but neither elucidated the beat-to-beat kinetics of Cam transport nor explained its link to mitochondrial ATP synthesis.

Numerous studies examining the relationship between mitochondrial transport of Na and Ca and ATP synthesis demonstrated that increase in Ca_m_ stimulates ATP production [64,67–69]. The Ca_m_ uniporter (MCU) accounts for the majority of Ca_m_ uptake, while NCLX the principal mechanism for Ca_m_ extrusion (Fig. 2) [70]. In series of rabbit cardiac mitochondria experiments Cox and Matlib [67] have studied the impact of Na_i_ on Ca_m_ and measured Ca_m_ by using fura-2. Incubating mitochondria with increasing concentrations of NaCl showed that decrease in Ca_m_ reduces state 3 respiration and NADH production. Inhibition of NCLX with three inhibitors of different potency (lowest to highest): d-*cis*-diltizam<clonazepam<CGP-37157 and the ruthenium red MCU inhibitor significantly increased Ca_m_, state 3 respiration and NADH production in a dose-dependent manner. This outcome suggested that Na_i_ overload impairs the matching between ATP supply and demand. However, isolated mitochondria experiments should be interpreted with caution, as the measurements are made in the absence of the key ATP utilizers including myosin ATPase, NKA and SERCA, intracellular compartments and metabolic pathways including glycolysis.

In isolated guinea pig cardiomyocytes Maack et al. [60] have shown that Na_i_ elevation significantly reduces both diastolic and systolic Cam. Consistently, the percentage of NAD(H) in the control group was maintained at ~62%, but in the high Na_i_ group was significantly reduced. Despite changes in Ca and [NADH], Na_i_ elevation did not change the mitochondrial membrane potential (Δψ_m_). Another premise of their work was that failure to maintain pyridine nucleotide reduction (NADPH) and in turn antioxidant flux due to high Na_i_ contributes to reactive oxygen species (ROS) overflow which is readily corrected by lowering Na_i_ in failing heart cells [71]. Collectively these results supported the argument that Na_i_ plays an important role in regulation of cardiac energy homeostasis. However, which metabolic pathways are most affected by Na_i_ overload and whether any of these changes were truly reflective of the regulatory mechanism in the beating heart remained to be shown.

A recent study by Hernansanz-Agustin et al. [72] has offered an additional mechanistic insight into the effect of Na on mitochondrial function and OXPHOS. Under the conditions of metabolic stress such as acute hypoxia which is typical of ischaemia/reperfusion injury, Na was shown to acts as a second messenger. Their study identified that hypoxia-mediated activation of Na/Ca_mito_ increases Na_mito_ and leads to release of Cam from calcium phosphate precipitates as well as Na-dependant reduction of the fluidity in the inner mitochondrial membrane. Consequently, alterations in the membrane fluidity caused a reduction in ubiquinone mobility between ETC II and III leads to ROS production. Inhibition of Na/Ca_mito_ was shown to block all these events.

## 6 Chronic and acute Na_i_ overload reprogram cardiac metabolism

In order to ensure that ATP supply matches consumption during increased cardiac contractility, mitochondria readily respond to increases in the cytosolic Ca [73]. Resultant rise in mitochondrial matrix calcium (Ca_mito_) activates key Ca-sensitive TCA cycle regulatory enzymes (CaDH_mito_) including pyruvate dehydrogenase (PDH), *α*-ketoglutarate dehydrogenase and the NAD-linked isocitrate dehydrogenase (Fig. 2) as well as ETC complex V [74] [68].

CaDH_mito_ activation leads to enhanced production of NADH, a critical intermediate for the electron transport chain (ETC) as well as redox signalling regulator (Fig. 2) [22].

This critical nexus of ATP supply-demand has been proposed to be affected by elevated Na_i_, which activates NCLX, decreasing Ca_m_ and leading to compromised NADH/NAD redox cycling and energetic inefficiency [22,75,76] (Fig. 2).

**Fig. 3:**
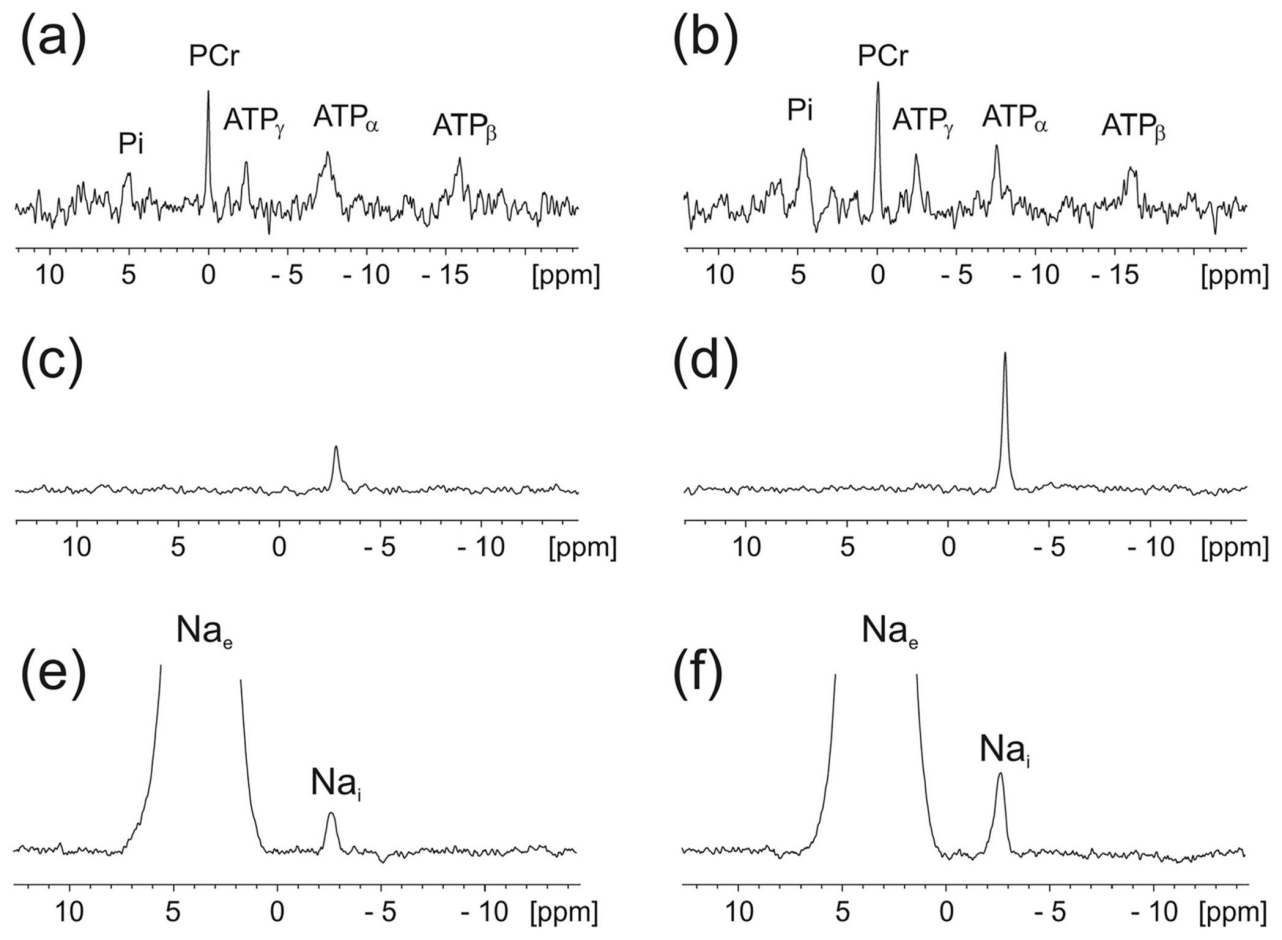
Representative ^31^P NMR, Triple Quantum Filtered ^23^Na and 1D ^23^Na NMR spectra from healthy and hypertrophied perfused mouse hearts (reproduced with permission from [9]). Perfused control heart spectra are shown in panels (a,c,e) and hypertrophied heart spectra in panels (b,d,f). NMR spectra were acquired using a Bruker Avance III 400 MHz wide-bore spectrometer [106] (a-b) ^31^P spectra, (c-d) triple quantum filtered (TQF) ^23^Na NMR spectra. (e-f) conventional single quantum ^23^Na NMR spectra acquired during infusion of 5 mM Tm(DOTP) shift reagent. (This figure was originally published in [9] and used with permission https://doi.org/10.1042/BST20170508).

**Fig. 4:**
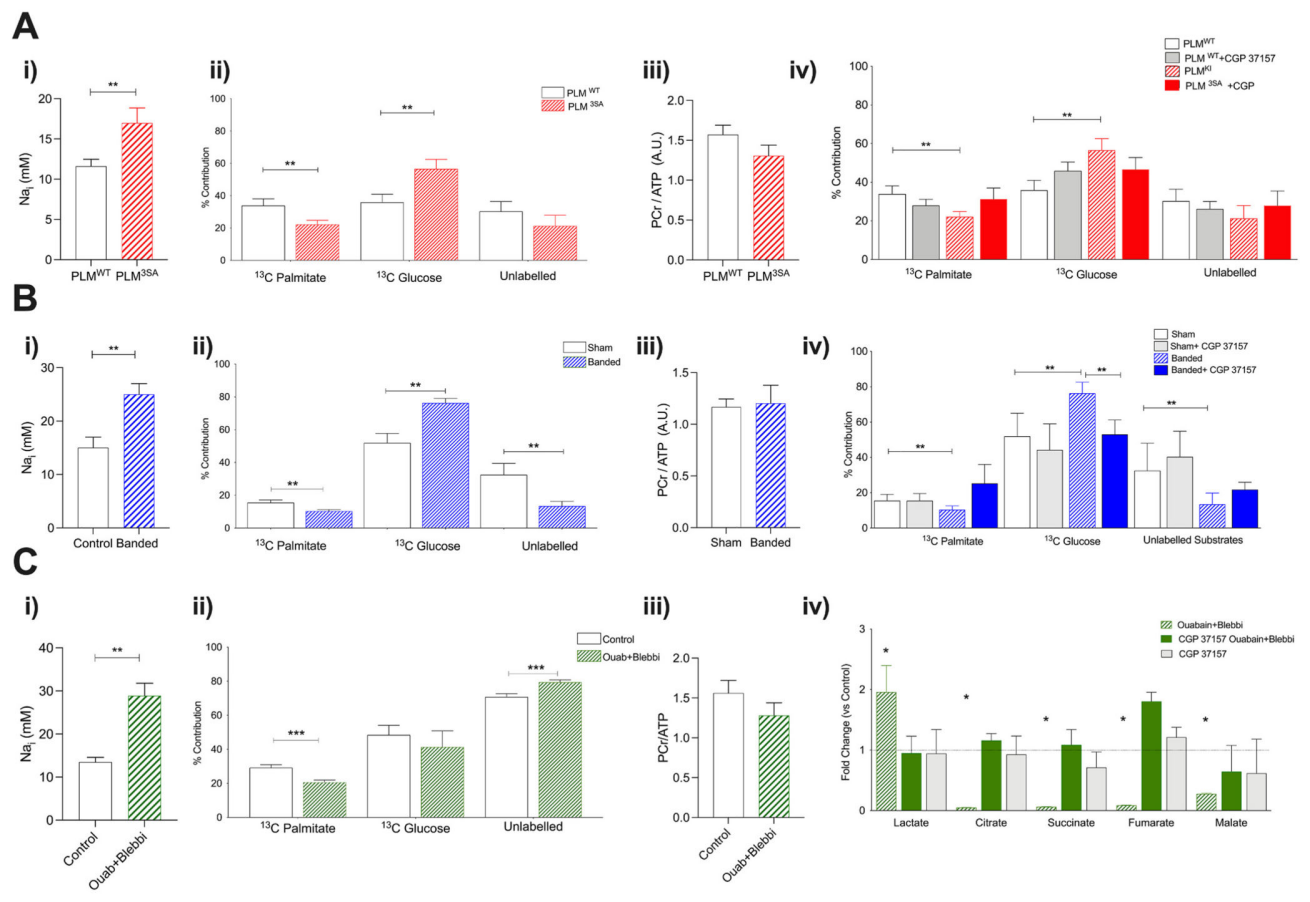
Elevation of intracellular sodium reprograms cardiac metabolism [43]. Chronic transgenic (A), chronic hypertrophy (B) and acute (C) myocardial sodium elevation precedes metabolic substrate switch (% contribution to oxidative metabolism A-C ii) without compromising cardiac energetics (^31^P NMR spectroscopy A-C iii). Myocardial substrate utilization was assessed by steady state ^13^C NMR spectroscopy (A-C ii). Acute (30 min) treatment with mitochondrial Na/Ca inhibitor CGP37157 ameliorates the substrate switch (A-C iv) as well as the metabolite depletion. (Figure based on data published in [43] and used with permission).

In our recently published work [43], we examined whether elevated myocardial Na_i_ regardless of its duration (30 min, 5-week or 25-week Na_i_ overload) or aetiology (pharmacological, transgenic or hypertrophy–mediated NKA inhibition, Fig. 4) results in myocardial metabolic derangement. Our study combined mouse hearts perfusions under physiological conditions including temperature and metabolic substrate availability with non-invasive in situ NMR spectroscopy: multiple quantum filtered ^23^Na to asses sodium, ^31^P to asses cardiac energetics (Fig. 3), steady-state ^13^C assessment of metabolic substrate flux and utilization. We combined our perfusion NMR experiments with ex vivo high-resolution ^1^H NMRs and LC GC MS/MS metabolomic profiling as well as with in silico modelling to examine the impact of elevated Na_i_ on mitochondrial ATP provision.

We observed that elevated Na causes common metabolic alterations that *precede* the onset of impaired energy reserve (PCr/ATP) and deterioration in function. This was observed in models of chronic and acute Na elevation: PLM^3SA^ mouse with NKA inhibition, left ventricular hypertrophy (TAC model) and pharmacological NKA inhibition with ouabain (Fig. 4). All these models shared a common ‘fingerprint’ of metabolic switch towards enhanced carbohydrate oxidation (Fig. 4). This switch appeared to be adaptive as high energy phosphate (PCr/ATP) and redox metabolite pools (NADH) were not compromised (Fig. 4).

However, given that we have observed depletion of the metabolic substrates including lactate as well as depletion of amino acids, it is possible that the metabolic adaptation observed in the long run may not be sufficient to maintain PCr/ATP ratio comparable to the healthy hearts. Mathematical simulations support this hypothesis based on flux analysis. However, additional tracer experiments are needed to assess flux distributions under these conditions. In silico work showed that the impact of elevated Na_i_ on mitochondrial ATP provision is mechanistically more complex than previous studies using isolated cells and organelles suggested. CardioNet mathematical modelling predicted maintenance of ATP supply by enhanced aerobic glucose metabolism and anaplerosis. The pivotal finding of our study was no evidence of impaired energetics - at least when Na overload is acute, or early in the hypertrophic process (Fig. 4). However, although PCr/ATP may not be impacted during early cardiac remodelling in our study, the kinetics of CK flux may still be disproportionately altered, as previously observed in HF patients [77]. Of note, acute CGP37157 inhibition (30 min) of NCLX attenuated all the changes in substrate utilization profile (palmitate vs glucose oxidation) as well as restored the levels of depleted metabolic intermediates (Fig. 4). Our results suggest that in a healthy heart this change in fuel supply can be switched on and off by simply changing cytosolic Na_i_ concentration. If this process is truly reversible, developing drugs that lower the elevated Na_i_ in HF will not only treat some of the EC coupling problems (i.e. diastolic dysfuntion) but may also improve the ATP supply.

In our perfused murine preparations glucose was the preferred substrate under physiological conditions. Whilst surprising, it is consistently observed that murine hearts perfused in isolation have a baseline preference for carbohydrates, potentially due to differences in oxygen needed to fully oxidize carbohydrates vs fatty acids (e.g., palmitate) [78–82]. Furthermore, the first-rate limiting step of glycolysis, hexokinase, operates under maximum velocity even at very low glucose concentrations (Km =0.02 mM), which ensures that the heart never runs out of fuel. Thus, flux increases for glycolysis in perfused hearts are possible under physiological conditions. What is clear from our studies is that compared to the prevailing baseline in isolated rat and mouse hearts, sodium elevation enhances glucose use and reduces fatty acid contribution over and above baseline.

## 7 Therapeutic potential

Since William Withering’s recognition of the efficacy of cardiac glycosides, Na_i_ has inadvertently been a HF therapeutic target for at least 200 years. Glycosides are not only present in plants such as the foxglove (*digitalis purpurea*), but are also endogenously found in animals under physiological conditions (e.g. ouabain, digoxin and bufalin) and are elevated in chronic kidney disease patients [83] and HF [84]. The positive inotropic effects of cardiac glycosides are well understood and involve NKA inhibition [85], the elevation of Na_i_ and the resetting of Na/Ca exchange leading to cellular Ca loading. While historically inotropic agents were considered to offer symptomatic relief to HF patients, it is relatively recently that this was recognised as ‘flogging a dead horse’ [86]. Recent clinical experience has shown that decreasing, rather than increasing, cardiac workload is a far more effective therapeutic strategy with pharmacological interventions such as beta blockers, diuretics (to reduce preload and afterload) and heart rate slowing agents (such as ivabradine) shown to have much improved outcomes [87]. Lowering intracellular sodium rather than elevating it, would therefore seem to be a desirable therapeutic aim in HF. As described, pathological Na elevation in the failing heart impairs metabolism, causes substrate switching, increases oxidative stress, increases cardiac work, impairs relaxation contributing to diastolic dysfunction, and is proarrhythmic - a plethora of reasons why it should be therapeutically targeted. Reducing Na influx with drugs like ranolazine or cariporide have been trialled with mixed results but since these target quantitatively small transmembrane Na fluxes (relative to the large efflux capacity of the NKA) targeting the NKA itself may be a more effective strategy [88,89]. The problem is that as yet we have no effective activators of the NKA.

While therapeutically reducing elevated Na may have multiple benefits, it may also be possible to target the consequences of Na elevation more directly and specifically to target deranged metabolism. In spite of the substantial data to support the pharmacological targeting of the substrate switch, successful translation to the clinic has been limited, with the exception, perhaps, of the cluster of drugs which target post-ischaemic myocardial energetic efficiency [90]. These include drugs targeting fatty acid oxidation inhibition via CPT1 such as etomoxir, ethyl-2-tetradecyl glycidate and oxfenicine [91,92]. However, etomoxir has the narrow therapeutic window, as in skeletal muscle CPT I inhibition leads to excess triglyceride accumulation and lipotoxicity [93]. Partial fatty-acid oxidation (PFox) inhibitors such as piperazine derivatives ranolazine and trimetazidine have been prescribed as anti-anginal drug in France [94]. They inhibit fatty-acid oxidation but also act indirectly by increasing the activity of pyruvate dehydrogenase to enhance glucose use. Trimetazadine is prescribed to DCM patients but only modestly improves cardiac function [95,96]. Pyruvate dehydrogenase kinase inhibition by sodium dichloroacetate improved contractile performance in HF patients, but a vehicle control group was omitted from the study design [97,98]. Therefore, given the currently limited success of targeting substrate metabolism, it is important to continue to investigate the therapeutic potential of alternative aspects of cardiac metabolism, including intermediary ATP supply pathways including intracellular NAD cycling [99,100]. Drugs targeting intermediary metabolism such as insulin-sensitizing (thiazolidinediones), lipid-lowering drugs (statins) and anaplerosis-targeting agents (propionyl l-carnitine) could also further help to elucidate the role these metabolic alterations play in transition from compensated hypertrophy to HF [101].

Given that mitochondrial Na/Ca exchange inhibitor CGP37157 would treat a consequence of sodium elevation rather than a primary cause, our recently published work suggests that reducing Na_i_ overload by pharmacological activation of NKA may be a more superior therapeutic strategy for attenuation of metabolic remodelling in HF with potentially fewer off-target effects [17,102]. Our study outcome also further underlines the unsuitability of cardiac glycosides such as digoxin for HF treatment as the elevation of Na_i_ would be expected to exacerbate pre-existent metabolic remodelling. Inhibitors of renal sodium-glucose co-transporter (SGLT2i) have been used at the successful type 2 diabetes treatment with cardioprotective, glycaemic control-independent effects observed in HF patients. It was widely postulated that SGLT2i produce their positive effects in HF by reducing Na_i_ by direct inhibition of cardiac NHE1 activity [103,104]. In our recently published study, we have used an integrated experimental approach at the cell (isolated rat ventricular myocytes cSNARF1 fluorescence imaging) and perfused organ level (perfused ^31^P and ^23^Na NMRS of the mouse, rat and guinea pig hearts) to assess the impact of SGLT2i empagliflozin on intracellular Na and NHE1. Our study has shown that SGLT2 inhibitors have no effect on Na_i_ concentration nor NHE1. This has been confirmed for the whole range of therapeutic empagliflozin doses and other SGLT2i’s. Therefore, any beneficial effects of SGLT2inhibitor family in failing hearts cannot be attributed to their action on myocardial NHE1 or intracellular Na [105].

Given the complexity of heart metabolism, it remains to be seen whether early prevention of myocardial Na_i_ elevation could either preclude the origin or change the course of metabolic remodelling in HF. This hypothesis warrants precision medicine approach and extensive further work including development of pharmacological agents that target these interconnected pathophysiological events.

## Figures and Tables

**Table 1:** Myocardial Na concentrations in healthy and failing hearts Adapted from [9].

Healthy Heart				Failing Heart			
Species	[Na]i(mM)	Assessment Technique		Species	[Na]_i_(mM)	Assessment Technique
Mouse	11.6	^23^Na NMR; perfused heart.	[106]	Mouse LVH	23	SBFI-loaded myocytes at rest.	[36]
	14	SBFI-loaded myocytes at rest.	[36]
Rat	12.7	Na-selective microelectrodes; muscle strips at 0.5 Hz.	[12]	Guinea pig LVH	12.1	Na-selective microelectrodes; muscle strips at rest.	[107]
	8.5–30	Na-selective microelectrodes; myocytes at rest.	[108,109]		12.8	^23^Na NMR; perfused heart.	[110]
Guinea pig	4.7–8.0	Na-selective microelectrodes; muscle strips at rest.	[107,111] [112–114],	Guinea pig Heart Failure	16.8	SBFI-loaded myocytes at rest.	[115]
	6.4	^23^Na NMR; perfused heart.	[110]
	5.1–5.2	SBFI-loaded myocytes at rest.	[10]
			[116]
Ferret	7.8	Na-selective microelectrodes; muscle strips at rest.	[117]	Ferret RVH	8.0	Na-selective microelectrodes; muscle strips at rest.	[117]
Rabbit	7.2	Na-selective microelectrodes; muscle strips at 0.5 Hz.	[11,12]	Human LVH	14.2	Na-selective microelectrodes; muscle strips at rest.	[107]
	3.8–4.5	SBFI-loaded myocytes at rest.	[11,118]	Human Heart Failure	12.1	SBFI-loaded muscle trips paced at 0.25 Hz.	[37]
	5.1–21	SBFI-loaded myocytes at rest.	[11,118] [10,119],	Human MVD	11.8	Na-selective microelectrodes; muscle strips at rest.	[107]
	17.5	^23^Na NMR; perfused arrested hearts.	[120]
Dog	8.9–10.4	Na-selective microelectrodes; Purkinje fibers at rest and 1 Hz.	[121]
Sheep	5–6.4	Na-selective microelectrodes; Purkinje fibers at 1 Hz and at rest.	[122,123]
	5.8–7.9	Na-selective microelectrodes; muscle strips at rest.	[114]
Human	8.0	SBFI-loaded muscle trips paced at 0.25 Hz.	[37]

## Abbreviations

EC: excitation-contraction
Na_i_: intracellular sodium
HF: heart failure
Ca: calcium (usually indicating Ca^2+^ ions in free solution)
Na: sodium (usually indicating Na^+^ ions in free solution)
Ca_mito_: intramitochondrial calcium
NMR: nuclear magnetic resonance
NKA: sodium/potassium ATPase
PLM: phospholemman
NCX: sarcolemmal sodium-calcium exchanger
NHE: sodium-proton exchanger
SERCA: sarcoendoplasmic reticulum Ca ATPase
FFA: free fatty acids
TCA: tricarboxylic acid cycle
ETC: electron transport chain
OXPHOS: oxidative phosphorylation
MCU: mitochondrial calcium uniporter
NCLX: mitochondrial sodium-calcium exchanger
Na_ex_: extramitochondrial sodium
SBFI: sodium-binding benzofuran isophthalate
Tm(DOTP): Thulium (III) 1,4,7,10-Tetraa-zacyclododecane-1,4,7,10-tetra(methylenephosphonate)
DCM: dilated cardiomyopathy.
